# Relationship between Serum Sirtuin 1 and Growth Hormone/Insulin-like Growth Factor 1 Concentrations in Children with Growth Hormone Deficiency and Idiopathic Short Stature

**DOI:** 10.3390/biomedicines12071433

**Published:** 2024-06-27

**Authors:** Anna Fedorczak, Dorota Kowalik, Justyna Kopciuch, Ewa Głowacka, Katarzyna Mikołajczyk, Marcin Tkaczyk, Andrzej Lewiński, Renata Stawerska

**Affiliations:** 1 Department of Endocrinology and Metabolic Diseases, Polish Mother’s Memorial Hospital—Research Institute, 93-338 Lodz, Poland; anna.fedorczak@outlook.com (A.F.); dorota.kowalik96@gmail.com (D.K.); alewin@csk.umed.lodz.pl (A.L.); 2 Center of Medical Laboratory Diagnostics and Screening, Polish Mother’s Memorial Hospital—Research Institute, 93-338 Lodz, Poland; justyna.kopciuch@iczmp.edu.pl (J.K.); ewa.biol@interia.pl (E.G.); 3 Department of Pediatrics, Immunology and Nephrology, Polish Mother’s Memorial Hospital—Research Institute, 93-338 Lodz, Poland; kasia.pazdziora@gmail.com (K.M.); marcin.tkaczyk@umed.lodz.pl (M.T.); 4 Department of Pediatrics, Nephrology and Immunology, Medical University of Lodz, 93-338 Lodz, Poland; 5 Department of Pediatric Endocrinology, Medical University of Lodz, 93-338 Lodz, Poland

**Keywords:** growth hormone, growth hormone deficiency, non-GH-deficient children, short stature, JAK/STATs pathway, insulin-like growth factor 1, IGF-1/IGFBP-3 molar ratio, sirtuin 1

## Abstract

Sirtuin 1 (SIRT1) inhibits growth hormone (GH) intracellular signaling for the insulin-like growth factor 1 (IGF-1) synthesis via the janus kinase (JAK)/signal transducer and activator of transcription proteins (STATs) pathway. The aim of this study was to compare SIRT1 concentrations in children with GH deficiency (GHD) and so-called idiopathic short stature (ISS, non-GH deficient), in order to determine the possible impact of changes in serum SIRT1 concentrations on the GH-IGF-1 axis. The study group included 100 short-stature children: 38 with GHD and 62 with ISS (maxGH in two stimulation tests <10 and ≥10 ng/mL, respectively). The control group consisted of 47 healthy, normal-height children. For each child, the concentrations of SIRT1, IGF-1 and insulin-like growth factor-binding protein 3 (IGFBP-3) were determined and the IGF-1/IGFBP-3 molar ratio was calculated. The level of SIRT1 was significantly higher in both groups of short children than in the controls (*p* < 0.0001), but there were no differences between GHD and ISS (mean ± SD: 0.89 ± 0.45 for ISS; 1.24 ± 0, 86 for GHD; and 0.29 ± 0.21 for controls). A significant negative correlation was found between SIRT1 and height standard deviation score (SDS), IGF-1 and IGF-1/IGFBP-3, but not between SIRT1 and maxGH. Elevated SIRT1 levels may serve as one of the mechanisms through which the secretion of IGF-1 is reduced in children with short stature; however, further research is required to confirm this issue.

## 1. Introduction

The regulation of growth processes in children is dependent on the actions of both the growth hormone (GH) and growth insulin-like growth factor 1 (IGF-1), wherein IGF-1 is the main mediator of GH activity for promoting longitudinal growth [1]. The activation of the Janus kinase 2 (JAK2)/Signal Transducers and Activators of Transcription (STATs) pathway is responsible for the synthesis of IGF-1 in hepatocytes in response to GH binding to its cell surface receptor [2]. Also, the IGF-binding protein 3 (IGFBP-3) gene is promoted through the same pathway [3]. The molar ratio of IGF-1 to IGFBP-3 is considered to be the indicator that best reflects GH activity in children [4,5].

Recently, it has been proven that sirtuin 1 (SIRT1), which is a NAD+-dependent deacetylase, is one of the modulators of JAK2/STATs pathway activity in hepatocytes [6]. SIRT1 is also involved in the cell cycle, apoptosis, response to oxidative stress, DNA repair, inflammatory processes and metabolism, as well as in the regulation of hunger and satiety [7,8,9,10]. Changes in SIRT1 expression depend on various factors and are intended to help maintain homeostasis. Generally, SIRT1 downregulates IGF-1 mRNA synthesis induced by GH [6]. Although SIRT1 acts primarily intracellularly, the serum SIRT1 concentration can also be determined. In our earlier study, we assessed the SIRT1 concentration in healthy children and analyzed the relationship between its concentration and gender, age and stage of puberty; we did not find correspondence between any of them [11]. Therefore, the assessment of the SIRT1 concentration in short-stature children seems interesting, as changes in its concentration may play an important role in growth disorders in children—being the cause or effect of certain abnormalities or adaptation mechanism. So far, reports on this issue are scarce [6]. Thus, the aim of the present study was to assess the serum SIRT1 concentrations in children with GH deficiency (GHD) (characterized by low GH concentration and low IGF-1 concentration) and in those with non-GH-deficient short stature (idiopathic short stature, ISS) (characterized by normal GH and low/normal IGF-1 concentration) in order to find a possible relationship between SIRT1 and GH levels (the latter assessed during stimulation tests), as well as between SIRT1 levels and IGF-1 and the IGF-1/IGFBP-3 molar ratio, depending on the diagnosis.

## 2. Materials and Methods

For this study, we enrolled 110 consecutive children with short stature who were admitted to the Department of Endocrinology and Metabolic Diseases of the Polish Mother’s Memorial Hospital—Research Institute (PMMH-RI) in Lodz, from January 2021 to April 2023, and whose parents consented to their participation in this study.

The inclusion criteria included the following: age, ≥2.0 and ≤17.0 years; patient’s height, less than −2.0 SDS with respect to age and sex; no identifiable organic or genetic causes of short stature; born at term (≥37 weeks of pregnancy) and with normal body length and weight (appropriate for gestational age, AGA); and the written consent of the legal representative to participate in this study. The height and weight of each child were measured using a Harpenden stadiometer and body eight scale, followed by the calculation of the BMI. The standard deviation score (SDS) for height, body weight and BMI were calculated according to the reference values for Polish children and adolescents [12,13]. As it was an analysis of children with short stature, in order to objectify the BMI SDS results, the height age (HA) of the children was calculated (as the age corresponding to the 50th percentile for a given child’s height), and the BMI SDS for the HA value was determined. From a clinical examination, the stage of pubertal development was assessed according to the Tanner scale [14]. The patients’ histories were taken, including their gestational age, birth weight and length, chronic diseases and other endocrine diseases, as well as parental height and family history. For the children with visible dysmorphic features, a karyotype assessment was performed. Children with other possible causes of short stature (e.g., celiac diseases (*n* = 1), hypothyroidism (*n* = 3), born small for gestational age (n = 6) and Turner syndrome (n = 1)) were excluded from this study (all together 11 children). Parental height data (measured at a visit or reported) were also collected and each patient’s target height (TH) was calculated, using the following formula: (mother’s height + father’s height)/2 + 6.5 cm for boys and (mother’s height + father’s height)/2 − 6.5 cm for girls. Next, the TH SDS value was also calculated using the same national reference values. Among the children with ISS, based on these data and the formula provided by Ranke et al., a group of patients with familial short stature (FSS) was distinguished—when height SDS > TH SDS − 1.28—and a group of non-FSS—when height SDS < TH SDS − 1.28 [15]. The patients with GHD underwent a pituitary MRI, which excluded organic causes of pituitary dysfunction and allowed for the diagnosis of idiopathic somatotropic hypopituitarism. The bone age (BA) was estimated according to Greulich–Pyle (G&P) evaluation standards, based on radiographs of the wrist and hand of the non-dominant hand.

During the hospital stay, two GH stimulation tests were conducted. The first test was performed after oral clonidine administration (at a dose of 0.15 mg/m^2^ of body surface area, with GH serum level measurements at the 0, 30th, 60th, 90th and 120th minute of the test). The second one was performed after an intramuscular glucagon injection (at a dose of 30 µg/kg of body mass, limited to a maximum of 1 mg), with the measurement of GH serum levels at the 0, 90th, 120th, 150th and 180th minute). For each child, before the first stimulation test, blood samples for SIRT1, IGF-1 and IGFBP-3 measurements were taken after nocturnal fasting, at 6:00 in the morning, approximately 10–12 h after the last meal.

The control group, presented in our previous study [11], consisted of 47 healthy children, matched by age and gender to the study group, with normal height and weight, hospitalized at PMMH-RI for follow-up examinations, but no abnormalities were found in them. For this group, the height, weight and puberty stage according to the Tanner scale, as well as the SIRT1, IGF-1 and IGFBP-3 concentrations were assessed.

All the analyses were conducted at the Centre for Medical Laboratory Diagnostics and Screening of the PMMH-RI. The methodology for the IGF-1, IGFBP-3 and GH determinations in the samples was described in great detail by our team in a previously published article [16]. The blood samples, in a volume of 2.6 mL for the SIRT1 evaluation, were drawn into S-monovette Serum Gel Cat tubes (SARSTEDT AG & Co. KG, Nümbrecht, Germany), and then the tubes were left at room temperature for 2 h to enable the samples to clot. Once the time had elapsed, the samples were centrifuged for 15 min at 1000× *g*, following the manufacturer’s recommendations for the kit that was used to examine the SIRT1 levels. The serum samples were visually assessed for hemolysis, and were then removed immediately and aliquoted into labeled Eppendorf 1.5 mL tubes. The samples were stored at −80 °C until the date of the assay. The SIRT1 concentration was determined by the double-binding ELISA method. For this purpose, 2 Human NAD-dependent deacetylase Sirtuin-1 (SIRT1/SIR2L1) ELISA Kits (Cusabio, Houston, TX, USA) were used. The steps were followed according to the manufacturer’s instructions (User Manual; catalogue number: CSB-E15058h). For the procedure, the initial step was the preparation of the samples and standards, and then applying them to the plate. The provided plate was pre-coated with SIRT1/SIR2L1-specific monoclonal antibodies. The preparatory step was followed by an incubation period of two hours at 37 °C. Upon the completion of this time, the wells were thoroughly and carefully drained of the fluid contents and without a washing procedure, biotin-conjugated antibodies were added. This was followed by another incubation period of an hour under the same conditions. As per the manufacturer’s instructions, three washing steps in total were applied, and blotting with a clean absorbent surface was also implemented. For the next step, streptavidin–horseradish peroxidase conjugate (HRP-avidin) was used and distributed into the wells and allowed to set for another incubation period of one hour at 37 °C. After that incubation ended, five wash steps in total were conducted. Following that action was the addition of chromogenic substrate 3,3′,5,5′-tetramethylbenzidine (TMB) and the incubation of the plate in the same temperature conditions for 20 min. The reaction was stopped with sulfuric acid, provided as a Stop Solution by the manufacturer. The last step was to read the absorbance using an ELISA plate reader (Bio-Rad iMark; Hercules, CA, USA) at 450 nm. The manufacturer ensures in the attached IFU that the sensitivity of the assay is 0.039 ng/mL, while the guaranteed detection range of the assay is 0.156 ng/mL–10 ng/mL, with an intra-assay coefficient of variation of less than 8%, and an inter-assay coefficient of variation of less than 10%.

The collected data underwent statistical analysis using STATISTICA ver. 13.3 software (Statsoft, Cracow, Poland). The normality of the distribution was assessed using the Shapiro–Wilk test, and the equality of variance was evaluated using Levene’s test. Non-parametric tests were used for intergroup comparisons of the quantitative continuous variables: the Kruskal–Wallis rank ANOVA and Mann–Whitney U test. Intergroup comparisons of the nominal/qualitative variables were performed using the chi-square test. A correlation analysis of the variables studied (Pearson correlation coefficient) was also conducted. The continuous variables were displayed as the mean and standard deviation (mean ± SD), and the categorical variables by N (%). Statistically significant differences were defined as *p*-values less than 0.05. Approval was obtained from the Bioethics Committee at the Polish Mother’s Memorial Hospital—Research Institute in Lodz (Opinion No. 47/2020).

## 3. Results

### 3.1. Study Group Characteristics

Finally, one hundred (100) children with short stature were enrolled in this study. GHD was diagnosed when the maximum GH secretion in both stimulation tests was found to be below 10 ng/mL, while ISS was diagnosed when the maximal GH secretion in at least one stimulation test was higher than or equal to 10 ng/mL. With respect to the results of the GH stimulation tests, the patients were divided as follow: 38 children were diagnosed with GHD and 62 children with ISS. The control group consisted of 47 healthy individuals with normal height. In the whole group, 86 (58.5%) children were boys, 61 (41.5%) were girls and their mean age was 10.45 ± 2.72 years. There were no statistically significant differences between the individual groups regarding sex or age. The characteristics of the study groups with respect to the diagnosis are presented in Table 1.

### 3.2. Results of Serum Tests

Since the division of the short-stature patients was based on the results of the GH peak in the stimulation tests, the statistical analysis obviously showed a significant difference (*p* < 0.0001) between the GH peak secretion in the GHD (GH peak < 10 ng/mL) and ISS (GH peak ≥ 10 ng/mL) groups (no stimulation tests were performed on the controls). In both (GHD and ISS) groups, the IGF-1 concentrations [(137.17 ng/mL ± 59.49) vs. (155.48 ng/mL ± 86.92) vs. (270.56 ng/mL ± 183.39), *p* < 0.0001)], and the IGF-1 SDS [(−1.67 ± 0.98) vs. (−1.28 ± 0.84) vs. (−0.39 ± 1.14), *p* < 0.0001)] were significantly reduced compared to the controls, and the IGF-1 SDS was significantly lower in the GHD than in the ISS group (*p* < 0.0386). Similarly, the IGFBP-3 levels [(3481 ng/mL ± 979) and (3482 ng/mL ± 979) vs. (4317 ng/mL ± 1435), *p* < 0.0017)] and the IGF-1/IGFBP-3 molar ratio [(0.22 ng/mL ± 0.06) and (0.23 ng/mL ± 0.09) vs. (0.32 ng/mL ± 0.16), *p* < 0.0088)] were found to be significantly lower in both groups of short children than in the control group, while they did not differ between the individual groups of short children (GHD and ISS), despite the completely different GH secretion and—therefore—final diagnosis. The results of the serum tests performed on the short-stature children and control group are presented in Table 2.

**Figure 1:**
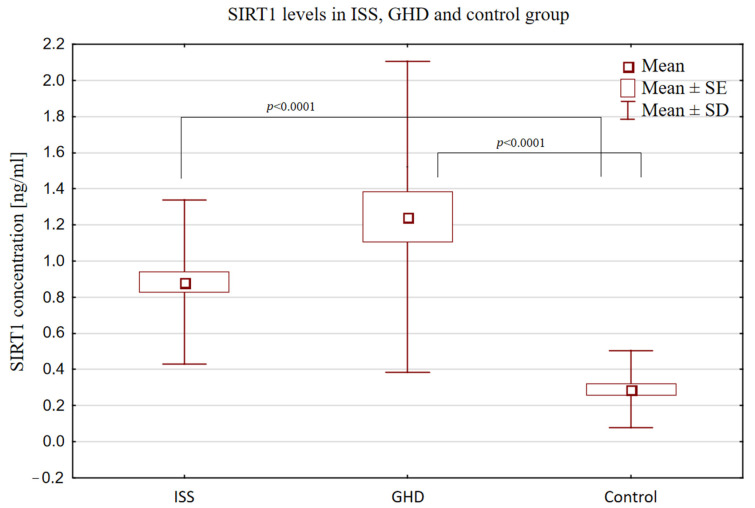
Comparison of SIRT1 concentrations in short children with ISS (GH secretion ≥ 10 ng/mL) and GHD (GH secretion < 10 ng/mL) and in the control group.

The SIRT1 concentration was significantly higher in both groups of short-stature children (GHD and ISS) than in the control group [(1.24 ng/mL ± 0.86) and (0.89 ng/mL ± 0.45) vs. (0.29 ng/mL ± 0.21), *p* < 0.0001)], but similarly to the IGF-1 and IGFBP-3 concentrations and the IGF-1/IGFBP-3 molar ratio value, it did not differ between the two groups of short children (GHD and ISS). The SIRT1 concentrations are displayed in Figure 1 to illustrate the wide range of results with respect to the mean in the GHD group.

### 3.3. Correlations of SIRT1 with Height, Body Mass and IGF-1

**Figure 2:**
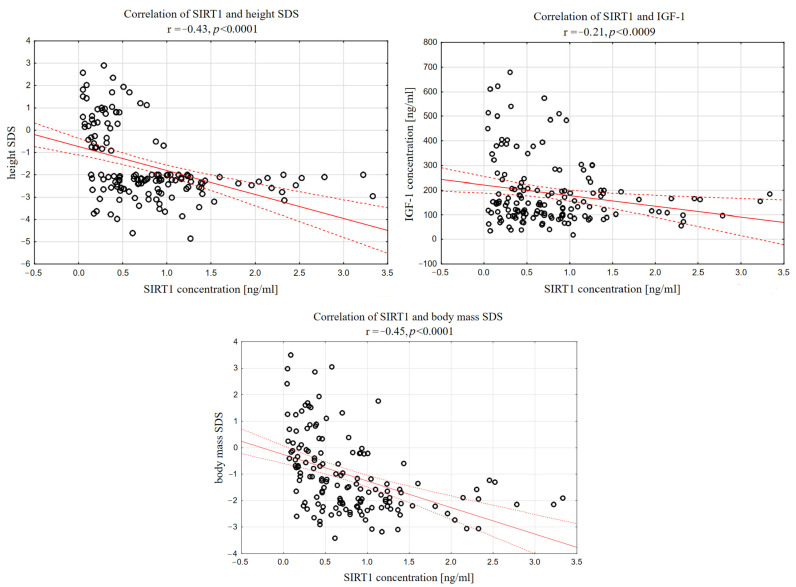
Correlations between sirtuin 1 and height SDS, IGF-1 and IGF-1/IGFBP-3 molar ratio in whole analyzed group of children.

In the whole analyzed group (short stature and control group), a significant negative correlation was found between SIRT1 and each of the listed parameters: height (r = −0.29, *p* < 0.0008), height SDS (r = −0.43, *p* < 0.0001), IGF-1 (r =−0.21, *p* < 0.0097), and IGF-1/IGFBP-3 molar ratio (r = −0.18, *p* < 0.0325). Notably, when we took a SIRT1 concentration of 1.5 ng/mL as the cut-off point, all the children whose SIRT1 concentration exceeded this value had a height SDS below <−2.0 and IGF-1 lower than 200 ng/mL (Figure 2).

**Figure 3:**
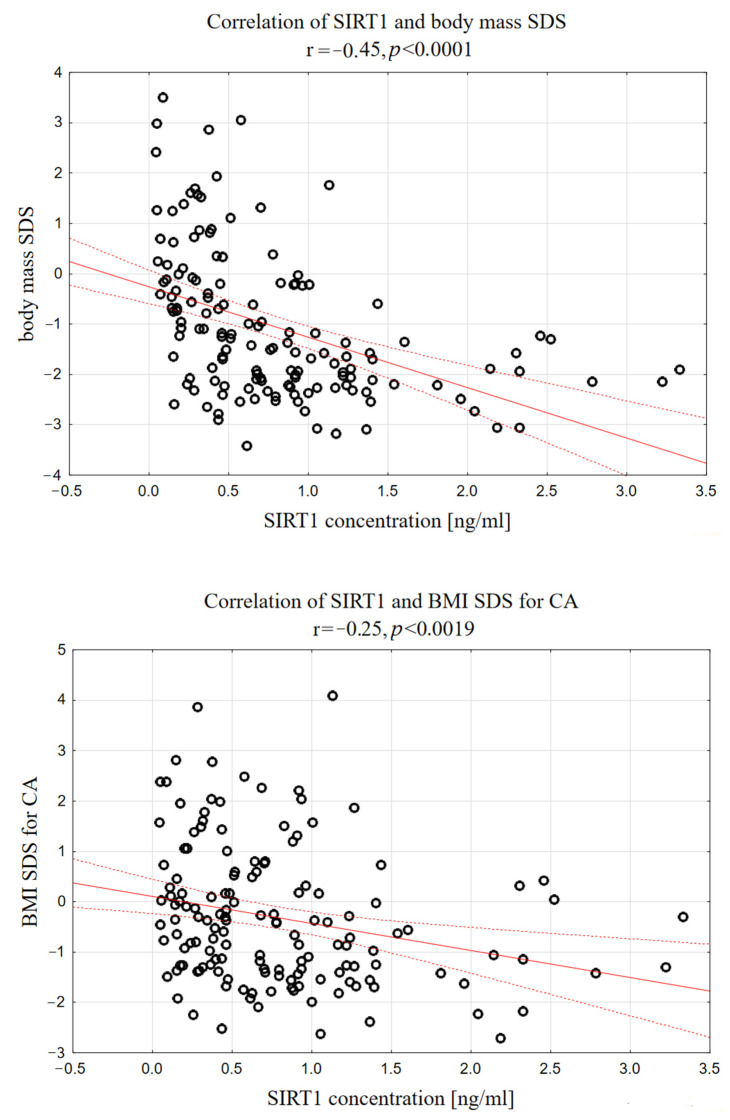
Correlations between serum SIRT1 concentration and body mass SDS, as well as serum SIRT1 and BMI SDS in analyzed group of short-stature children.

Moreover, serum SIRT1 correlated negatively with body mass (r = −0.29, *p* < 0.0005), body mass SDS (r = −0.42, *p* < 0.0001), as well as BMI (r = −0.24, *p* < 0.0060) and BMI SDS (r = −0.26, *p* < 0.0019), as shown in Figure 3.

As SIRT1 does not have a normal distribution, log-transformed SIRT1 values were also calculated, revealing significant correlations between the LogSIRT1 and the following parameters: height (r = −0.35, *p* < 0.0001), height SDS (r = −0.57, *p* < 0.0001), IGF-1 (r = −0.27, *p* < 0.0009), IGF-1/IGFBP-3 molar ratio (r = −0.26, *p* < 0.0025), body mass (r = −0.36, *p* < 0.0001), body mass SDS (r = −0.59, *p* < 0.0001), as well as BMI (r = −0.25, *p* < 0.0027) and BMI SDS (r = −0.28, *p* < 0.0007).

### 3.4. Sirtuin 1 Levels with Respect to the Severity of GHD

In the whole group of short-stature children, no correlations between SIRT1 and the serum GH peak in any of the stimulation tests were observed. However, after dividing them into subgroups, SIRT1 was positively correlated with the max GH secretion in the stimulation tests only in GHD group (r = 0.37, *p* < 0.05). Moreover, in the GHD group, the BMI SDS was correlated negatively with the max GH secretion (r = −0.43, *p* < 0.05). So, thinner children with GHD had higher GH secretion and higher sirtuin 1 levels.

Subsequently, the short-stature children were divided into three subgroups with respect to their maximal GH secretion in both stimulation tests: severe GHD (sGHD), when maxGH was <7 ng/mL (n = 16); partial GHD (pGHD), when maxGH was in the range of 7–10 ng/mL (n = 22); and ISS, when GH max was ≥10 ng/mL (n = 62). There were only four children with very low GH secretion (<3 ng/mL), so they were classified as sGHD. The auxological parameters and serum test results of the above-subdivided groups are presented in Table 3 and Table 4.

**Figure 4:**
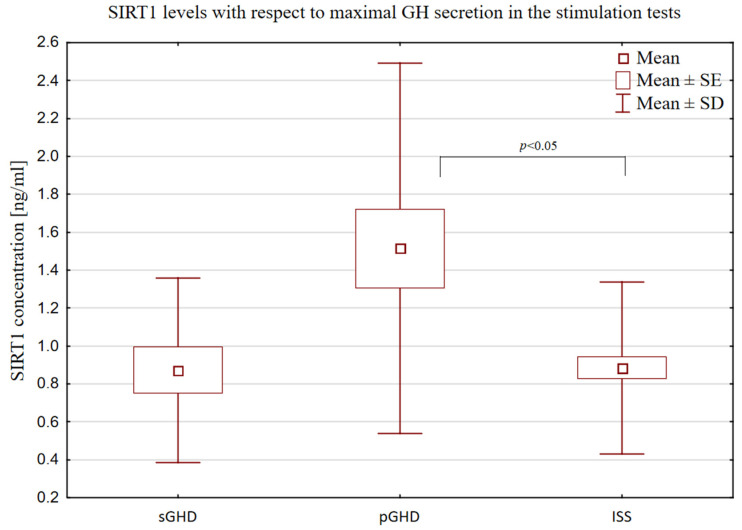
Comparison of SIRT1 levels in ISS (GH secretion ≥ 10 ng/mL), pGHD (GH secretion ≥ 7 and <10 ng/mL) and sGHD (GH secretion < 7 ng/mL) groups.

The SIRT1 levels were significantly higher in the patients with pGHD than in the patients with ISS [(1.51 ng/mL ± 0.98) vs. (0.89 ng/mL ± 0.45), *p* < 0.0391, Figure 4]. Children with sGHD formed a very small group (n = 16), but it became visible that the SIRT1 levels were the lowest in them (0.87 ng/mL ± 0.49), and did not differ from the SIRT1 levels observed in the ISS group. In turn, the ISS and pGHD groups were similar in terms of their BMI SDS (−0.87 ± 1.08 and −0.59 ± 1.09) and IGF-1 SDS (−1.28 ± 0.84 and −1.44 ± 0.89), and differed significantly from the sGHD group (BMI SDS 0.79 ± 1.09, *p* < 0.0020; IGF-1 SDS −1.99 ± 1.04, *p* < 0.0395) in this respect.

We also compared the children with ISS according to a diagnosis of familial short stature (FSS) or non-FSS, but we found no correspondence with SIRT1 levels.

## 4. Discussion

Based on the results of our research, we found that the concentration of SIRT1 in serum was significantly higher in both groups of short-stature children (ISS and GHD) than in the control group. Thus, it seems that SIRT1 may play a role in growth disorders in children. However, the mechanisms involved in these relationships are not clear.

As is known, short stature and a slow growth rate in children depend primarily on a low concentration or availability of IGF-1. A reduced concentration of IGF-1 may result from a GH deficiency, since the GH is the most important stimulator of its secretion [17]. However, many other causes of insufficient IGF-1 secretion are also known. These include chronic diseases (e.g., of the respiratory or circulatory system), liver dysfunction, malabsorption, malnutrition and other endocrine diseases, such as hypothyroidism [18]. In our research, at the initial stage, we eliminated cases of children suffering from chronic diseases, complaining of gastrointestinal problems, and children with untreated hypothyroidism. A history was taken of the patients, which did not point to undernutrition as a clinically evident cause of short stature. However, we did not exclude patients with low BMI from this study (as this was the majority of them). Therefore, it was difficult to determine whether the children who were poor eaters and whose caloric balance was negative, for example, in relation to proteins, were not qualified for the study group. Nevertheless, in the group of children we analyzed, the causes of IGF-1 decrease with normal GH levels remained uncertain. We believe that at least some of them may be related to excessive SIRT1 levels.

Our study shows significant inverse relationships between the SIRT1 concentration and the IGF-1 levels, the IGF-1/IGFBP-3 molar ratio and the degree of growth deficiency. It appears that in certain adverse conditions, increased SIRT1 levels may serve as a factor leading to the inhibition of IGF-1 secretion to conserve energy and maintain homeostasis. It should be emphasized that SIRT1 is involved in reacting to metabolic imbalances caused by caloric restriction and malnutrition [11,19]. We found that SIRT1 levels were related to a child’s nutritional status. Within our investigation, SIRT1 levels were negatively correlated with the weight SDS and BMI SDS. Correspondingly, *SIRT1* gene expressions were also upregulated in response to calorie restriction and weight loss, as well as negatively correlated with BMI [20]. It is known that GH has a hyperglycemic effect by releasing glucose reserves from stores, whereas IGF-1 has a hypoglycemic effect similar to insulin [21,22,23]. Therefore, in cases of malnutrition conditions, an increased secretion of GH from the pituitary and decreased secretion of IGF-1 from the liver at the same time (as, for example, in anorexia nervosa) is beneficial for the body (to maintain glucose levels) [24]. It seems that an increasing concentration of SIRT1 contributes to this, as SIRT1 has been found to negatively regulate GH-induced IGF-1 production by modulating JAK2/STATs pathway activity in hepatocytes [6,25]. Whether such a prolonged status could cause growth impairment (in response to an insufficient IGF-1 concentration, with normal or even high GH levels) is a subject for further research. Similarly, an inverse association between SIRT1 and IGF-1 has also been observed in cases of intrauterine growth restriction (IUGR). Chriet et al. [20] found that the trajectories of gene expression for sirtuins and metabolic genes were perturbed in pigs with IUGR, showing a correspondence with IGF-1 dysregulation. There was a notable increase in *SIRT1* gene expression accompanied by decreased levels of IGF-1.

On the other hand, the reason why SIRT1 levels were higher in the children with GHD (both pGHD and sGHD) than in the control group is not fully understood. Our results also reveal that SIRT1 was positively correlated with the maximum GH secretion in the stimulation tests only in the GHD group. This might be explained in the following way. In children with GHD and a normal BMI, there is no limitation to providing exogenous nutrients (food), but because of the primarily low secretion of GH, there is an impairment in the catabolic actions of GH—providing glucose and free fatty acids from tissues into the bloodstream. Such a constellation is supposed to trigger signals similar to the caloric restriction state (activating ghrelin) and—in this way—activate SIRT1 in some tissues, e.g., the hypothalamus. Thus, we speculate that in the case of patients with GHD, SIRT1 may increase in response to decreased GH (as an attempt to promote GH secretion in a positive feedback mechanism). Actually, SIRT1 is supposed to influence GH signal transduction at various levels [26]. SIRT1 is widely expressed in the hypothalamus (in particular in the arcuate nucleus) [27]. Moreover, SIRT1 activity in the brain is probably involved in the regulation of the somatotropic axis in response to energy supply [28]. It has been proven that in the arcuate nucleus (ARC), the majority of the GH receptor-expressing neurons also express SIRT1, and respond to fasting by upregulating *SIRT1* expression [28,29]. It is also worth mentioning ghrelin, which specifically triggers a central SIRT1/p53 pathway—essential for its orexigenic action [30].

Although the statistical analysis did not show a significant difference between the SIRT1 concentration in the GHD and ISS groups (*p* < 0.09), it is worth emphasizing that among the children in the GHD group, there were cases of very high SIRT1 concentrations (up to 3.33 ng/mL), and the dispersion around the mean was large, which indicates the heterogeneity of this group. The division of patients into sGHD (GH secretion < 7 ng/mL) and pGHD (GH secretion 7–10 ng/mL) also sheds some light on this issue. Children with pGHD presented with similar features to the ISS group—they were similarly thin and had comparably decreased levels of IGF-1 (despite different GH secretion), and significantly differed from the patients with severe GHD in those respects.

This leads to the question of whether patients with pGHD overlap or form the same group as patients with ISS. It is often mentioned in medical reports that children with pGHD may constitute a group that does not require rhGH treatment and does not differ significantly from children with ISS [31,32]. In fact, distinguishing between GHD and ISS raises difficulties, as there is a continuum between normal GH secretion and GH deficiency [33]. To diagnose GHD, the maximum secretion of GH in stimulation tests has been arbitrarily assumed to be less than 10 ng/mL (or less than 7 depending on the recommendations) [34]. Patients with higher GH secretion are diagnosed with ISS. Such an arbitrary division is questionable, especially given the poor reproducibility and reliability of the stimulation tests. Although at present there are no better tools for diagnosing GHD than the stimulation tests, this topic should be the subject of further research and discussion. Nevertheless, the levels of SIRT1 differed significantly between these two groups (pGHD and ISS). At present, it is difficult to determine the reason for this observation; one can only speculate that the higher levels of SIRT1 in pGHD may primarily result from decreased GH secretion—and that differences in GH secretion levels do matter.

It should also be noted that although SIRT1 is an intracellular enzyme, it is also found in the serum and its extracellular pool appears to play a role in the human body [11]. However, the receptors for SIRT1 have not been described, and how SIRT1 is transported in the blood has not been determined yet. To date, only one study has assessed SIRT1 serum levels in terms of growth. It did not reveal higher SIRT1 levels in the short-stature boys than in the controls, but the GH secretion status in those children was not reported, and one group had been already treated with rhGH [35]. We believe that the results of our study will contribute to the knowledge on this topic—as we have shown that the serum concentration of SIRT1 may reflect its intracellular biological function [6].

Increased SIRT1 levels in children with short stature may lead to favorable metabolic effects on the human body [36,37]. Moreover, in numerous studies, SIRT1 activation has been shown to regulate pathways with beneficial effects on aging and metabolic disorders, inflammatory processes, DNA damage and oxidative stress [38,39,40,41,42]. Our analysis, which is one of the first attempts to assess serum SIRT1 concentrations in children, displays a link between short stature, GH and SIRT1. The involvement of SIRT1 in the regulatory mechanisms concerning growth, metabolism and lifespan provides an interesting perspective for further research.

## 5. Conclusions

In conclusion, in short-stature children, regardless of GH secretion, SIRT1 serum concentrations were increased. Considering the significant negative correlations between SIRT1 concentration and the levels of IGF-1, the IGF-1/IGFBP-3 molar ratio and the severity of the growth deficiency, elevated SIRT1 levels may serve as one of the mechanisms through which the secretion of IGF-1 is reduced in children with short stature; however, further research is required to confirm this issue.

## Figures and Tables

**Table 1:** Study groups characteristics with respect to diagnosis (numerical variables are given as means ± SD and min; max range).

Variable		ISS, n = 62	GHD, n = 38	ISS vs. GHD, *p*<	Controls, n = 47	*p*<
age [years]		10.4 ± 2.75	10.75 ± 2.88	0.4754	10.35 ± 2.6	0.6780
		5.02; 15.98	3.01; 15.26		4.21; 14.35	
sex, N (%)	female	28 (45.16%)	13 (34.21%)	0.2798	20 (42.5%)	0.5500
	male	34 (54.84%)	25 (65.7%)		27 (57.5%)	
height [cm]		127.93 ± 14.17	130.97 ± 15.39	0.2233	146.46 ± 17.89	0.0001 *
		94; 150.1	89; 154.4		105; 181	
height SDS		−2.62 ± 0.51	−2.40 ± 0.3	0.1586	0.52 ± 1.02	0.0001 *
		−4.85; −2	−3.77; −2		−1.07; 2.91	
body mass [kg]		26.56 ± 7.86	31.17 ± 10.54	0.0285 *	39.40 ± 14.25	0.0001 *
		12.2; 44.6	12.5; 54.2		16; 78	
body		−1.99 ± 0.62	−1.40 ± 1.05	0.0050 *	0.48 ± 1.26	0.0001 *
mass [SDS]		−3.41; −0.60	−3.05; −1.77		−2.08; 3.5	
BMI [kg/m^2^]		15.82 ± 2.13	17.61 ± 3.22	0.0060 *	17.69 ± 2.73	0.0004 *
		12.53; 22.87	12.93; 25.89		13.83; 24.07	
BMI SDS for		−0.87 ± 1.08	−0.01 ± 1.61	0.0066 *	0.18 ± 1.27	0.0001 *
CA		−2.60; 2.27	−2.69; 2.46		−2.25; 2.83	
height age,		7.77 ± 2.35	8.33 ± 2.45	0.2062	x	x
HA [years]		2.79; 12.81	2.96; 11.82			
BMI SDS for		−0.34 ± 1.36	0.69 ± 1.83	0.0057 *	x	x
HA		−2.70; 4.60	−1.98; 5.03			

* *p* < 0.05 is bolded. ISS—idiopathic short stature; GHD—growth hormone deficiency; CA—calendar age; SDS—standard deviation score; BMI—body mass index; HA—height age.

**Table 2:** The results of the serum tests performed on the study groups (GHD and ISS) and on the control group (numerical variables are presented as the means ± SD and min; max range).

Variable	ISS, n = 62	GHD, n = 38	ISS vs. GHD, *p*<	Control Group, n = 47	*p*<
Max GH after	13.98 ± 4.42	6.05 ± 2.64	0.0001 *	x	x
clonidine [ng/mL]	3.30; 26.12	0.90; 9.83			
Max GH after	9.94 ± 5.48	4.91 ± 2.66	0.0001 *	x	x
glucagon [ng/mL]	1.88; 27.25	0.28; 9.85			
IGF-1 [ng/mL]	155.48 ± 86.92	137.17 ± 59.49	0.6443	270.56 ± 183.39	0.0001 *
	40.00; 510.90	19.10; 303.90		36.60; 679.40	
IGF-1 SDS	−1.28 ± 0.84	−1.67 ± 0.98	0.0386 *	−0.39 ± 1.14	0.0001 *
	−2.92; 0.83	−3.69; 0.29		−3.67; 1.41	
IGFBP-3	3482 ± 979	3481 ± 979	0.7493	4317 ± 1435	0.0017 *
[ng/mL]	2100; 5521	1458; 5703		1542; 6336	
IGF-1/IGFBP-3	0.23 ± 0.09	0.22 ± 0.06	0.5820	0.32 ± 0.16	0.0088 *
molar ratio	0.08; 0.51	0.06; 0.42		0.11; 0.71	
SIRT1 [ng/mL]	0.89 ± 0.45	1.24 ± 0.86	0.090	0.29 ± 0.21	0.0001 *
	0.15; 2.14	0.16; 3.33		0.04; 0.96	

* *p* < 0.05 is bolded. GH—growth hormone; ISS—idiopathic short stature; GHD—growth hormone deficiency; CA—calendar age; IGF-1—insulin-like growth factor 1; SDS—standard deviation score; IGFBP-3—IGF-binding protein 3; m.r.—molar ratio; SIRT1—sirtuin 1, x—test was not performed.

**Table 3:** Comparison of auxological parameters in ISS, pGHD and sGHD groups (numerical variables are presented as mean ± SD and min; max range).

Variable		ISS, n = 62	pGHD, n = 22	sGHD, n = 16	*p*<
age [years]		10.4 ± 2.75	10.34 ± 2.84	11.32 ± 2.92	0.6780
		5.02; 15.98	5.35; 13.85	3.01; 15.26	
sex, N (%)	female	28 (45.16%)	9 (40.91%)	12 (75%)	0.5500
	male	34 (54.84%)	13 (59.09%)	5 (25%)	
height [cm]		127.93 ± 14.17	128.59 ± 15.05	134.25 ± 15.73	0.2134
		94; 150.1	101.20; 148.50	89; 154.40	
height SDS		−2.62 ± 0.64	−2.46 ± 0.56	−2.32 ± 0.48	0.3321
		−4.85; −2	−3.77; −2	−2.61; −2.09	
BMI [kg/m^2^]		15.82 ± 2.13 **a,b**	16.36 ± 2.25 **a,c**	19.33 ± 3.60 **b,c**	0.0011 *
		12.53; 22.87	12.93; 21.48	13.89; 25,89	
BMI SDS		−0.87 ± 1.08 **a**	−0.59 ± 1.09 **b**	0.79 ± 1.09 **a,b**	0.0020 *
for CA		−2.60; 2.27	−2.17; 1.52	2.17; 1.52	
height age,		7.77 ± 2.35	7.97 ± 2.44	8.83 ± 2.45	0.2288
HA [years]		2.83; 12.81	3.75; 11.5	2.16; 12	
BMI SDS		−0.34 ± 1.36 **a**	−0.01± 1.26	1.65 ± 2.08 **a**	0.0018 *
for HA		−2.70; 4.60	−1.98; 2.53	−1.87; 5.03	

* The values in the columns marked with the same bold letters (a, b, c) differ significantly; *p* < 0.05. GH—growth hormone; ISS—idiopathic short stature; pGHD—partial growth hormone deficiency; sGHD—severe growth hormone deficiency; CA—calendar age; SDS—standard deviation score; BMI—body mass index; HA—height age.

**Table 4:** Sirtuin 1 levels in short-stature subjects in groups with respect to maximal growth hormone secretion in stimulation tests.

Variable	ISS, n = 62	pGHD, n = 22	sGHD, n = 16	*p*<
GH peak	15.23 ± 4.28 **a,b**	8.52 ± 0.98 **a,c**	4.3 ± 2.19 **b,c**	0.0001 *
[ng/mL]	10; 27.25	7.09; 9.85	0.90; 6.9	
IGF-1	155.48 ± 86.92	137.72 ± 56.35	136.43 ± 65.45	0.8942
[ng/mL]	40.00; 510.90	55.40; 287.20	19.10; 303.90	
IGF-1 SDS	−1.28 ± 0.84 **a**	−1.44 ± 0.89	−1.99 ± 1.04 **a**	0.0395 *
	−2.92; 0.83	3.63; 0.29	−2.61; −1.40	
IGFBP-3	3482 ± 979	3501 ± 935	3454 ± 1069	0.9379
[ng/mL]	2100; 5521	2180; 5703	1458; 5628	
IGF-1/IGFBP-3	0.23 ± 0.09	0.22 ± 0.07	0.21 ± 0.06	0.8570
	0.08; 0.51	0.11; 0.42	0.06; 0.30	
SIRT1 [ng/mL]	0.89 ± 0.45 **a**	1.51 ± 0.98 **a**	0.87 ± 0.49	0.0391 *

* The values in the columns marked with the same bold letters (a, b, c) differ significantly; *p* < 0.05. GH—growth hormone; ISS—idiopathic short stature; pGHD—partial growth hormone deficiency; sGHD—severe growth hormone deficiency; CA—calendar age; SDS—standard deviation score; BMI—body mass index; HA—height age.

## Data Availability

The data presented in this study are available on request from the corresponding author.
